# Respiratory symptoms in adults are related to impaired quality of life, regardless of asthma and COPD: results from the European community respiratory health survey

**DOI:** 10.1186/1477-7525-8-107

**Published:** 2010-09-27

**Authors:** Marianne Voll-Aanerud, Tomas ML Eagan, Estel Plana, Ernst R Omenaas, Per S Bakke, Cecilie Svanes, Valerie Siroux, Isabelle Pin, Josep M Antó, Benedicte Leynaert

**Affiliations:** 1 Institute of Medicine, University of Bergen, Bergen, Norway; 2 Centre for Research in Environmental Epidemiology (CREAL), Barcelona, Spain; 3 Department of Thoracic Medicine, Haukeland University Hospital, Bergen, Norway; 4 Centre for Clinical Research, Haukeland University Hospital, Bergen, Norway; 5 CHU de Grenoble, INSERM U823, Université Joseph Fourier-Grenoble, Grenoble, France; 6 Municipal Institute of Medical Research (IMIM-Hospital del Mar); CIBER Epidemiologia y Salud Pública (CIBERESP); and Universitat Pomeu Fabra, Barcelona, Spain; 7 Institut National de la Santé et de la Recherche Médicale, Unit 700, Epidemiologie, Paris, France

## Abstract

**Background:**

Respiratory symptoms are common in the general population, and their presence is related to Health-related quality of life (HRQoL). The objective was to describe the association of respiratory symptoms with HRQoL in subjects with and without asthma or COPD and to investigate the role of atopy, bronchial hyperresponsiveness (BHR), and lung function in HRQoL.

**Methods:**

The European Community Respiratory Health Survey (ECRHS) I and II provided data on HRQoL, lung function, respiratory symptoms, asthma, atopy, and BHR from 6009 subjects. Generic HRQoL was assessed through the physical component summary (PCS) score and the mental component summary (MCS) score of the SF-36.

Factor analyses and linear regressions adjusted for age, gender, smoking, occupation, BMI, comorbidity, and study centre were conducted.

**Results:**

Having breathlessness at rest in ECRHS II was associated with mean score (95% CI) impairment in PCS of -8.05 (-11.18, -4.93). Impairment in MCS score in subjects waking up with chest tightness was -4.02 (-5.51, -2.52). The magnitude of HRQoL impairment associated with respiratory symptoms was similar for subjects with and without asthma/COPD. Adjustments for atopy, BHR, and lung function did not explain the association of respiratory symptoms and HRQoL in subjects without asthma and/or COPD.

**Conclusion:**

Subjects with respiratory symptoms had poorer HRQoL; including subjects without a diagnosis of asthma or COPD. These findings suggest that respiratory symptoms in the absence of a medical diagnosis of asthma or COPD are by no means trivial, and that clarifying the nature and natural history of respiratory symptoms is a relevant challenge.

Several community studies have estimated the prevalence of common respiratory symptoms like cough, dyspnoea, and wheeze in adults [1-3]. Although the prevalence varies to a large degree between studies and geographical areas, respiratory symptoms are quite common. The prevalences of respiratory symptoms in the European Community Respiratory Health Study (ECRHS) varied from one percent to 35% [1]. In fact, two studies have reported that more than half of the adult population suffers from one or more respiratory symptoms [4,5].

Respiratory symptoms are important markers of the risk of having or developing disease. Respiratory symptoms have been shown to be predictors for lung function decline [6-8], asthma [9,10], and even all-cause mortality in a general population study [11]. In patients with a known diagnosis of asthma or chronic obstructive pulmonary disease (COPD), respiratory symptoms are important determinants of reduced health related quality of life (HRQoL) [12-15]. The prevalence of respiratory symptoms exceeds the combined prevalences of asthma and COPD, and both asthma and COPD are frequently undiagnosed diseases [16-18]. Thus, the high prevalence of respipratory symptoms may mirror undiagnosed and untreated disease.

The common occurrence of respiratory symptoms calls for attention to how these symptoms affect health also in subjects with no diagnosis of obstructive airways disease. Impaired HRQoL in the presence of respiratory symptoms have been found in two population-based studies [6,19], but no study of respiratory sypmtoms and HRQoL have separate analyses for subjects with and without asthma and COPD, and no study provide information about extensive objective measurements of respiratory health.

The ECRHS is a randomly sampled, multi-cultural, population based cohort study. The ECRHS included measurements of atopy, bronchial hyperresponsiveness (BHR), and lung function, and offers a unique opportunity to investigate how respiratory symptoms affect HRQoL among subjects both with and without obstructive lung disease.

In the present paper we aimed to: 1) Describe the relationship between respiratory symptoms and HRQoL in an international adult general population and: 2) To assess whether this relationship varied with presence of asthma and/or COPD, or presence of objective functional markers like atopy and BHR.

## Methods

### Study population

The ECRHS I [20] was undertaken in 1991 through 1993, and comprised representative population samples of adults aged 20-44 years in 48 centres in 22 countries. Altogether 137 619 subjects completed a postal questionnaire on respiratory health, giving a median response rate of 78% (stage I). Randomly selected representative subsamples of subjects from 36 centres participated in interviews and standardised clinical testing (ECRHS I stage II). The ECRHS II [21] was a follow-up study of respondents to ECRHS I stage II, conducted from 1998 through 2002. Of the invited, a total of 11 168 responded (response rate 76%). The respondents completed interviews and standardised clinical testing with blood tests, spirometry, and methacholine challenge. A total of 6009 subjects from 24 centres in 11 countries completed data regarding respiratory symptoms, HRQoL, and lung function in the ECHRS II. The regional ethics committees approved the study, and all participants signed informed consent. The full protocol is available at http://www.echrs.org.

### Questionnaire information

Information on respiratory symptoms was obtained in ECRHS II by questions 1-11 listed in appendix 1. Asthma was defined as ever having had asthma, question 12 in appendix 1 (ECRHS II).

Smoking was categorized as never-, ex- and current, where the current smokers were the ones that reported having smoked during the last month in the ECRHS II. The longest held job during the follow up period was used as a marker of socioeconomic status, and classified into 5 groups as 1) managers and professionals; 2) technicians; 3) other non-manual workers; 4) manual workers; and 5) non-classified workers. Data on comorbidities were obtained by the questions listed in appendix 2. Presence of comorbidity was included as a dichotomized variable.

The Short Form-36 (SF-36) questionnaire was used to assess HRQoL in the ECRHS II. The SF-36 is a standardized, widely used and validated general HRQoL questionnaire, which is translated into several languages [22]. It includes 36 questions from which the physical component summary (PCS) and the mental component summary (MCS) were computed as described in the SF-36 User's Manual [23]. The scores were transformed so that the mean score was 50 and the standard deviation was 10 [24]. A higher score indicates better quality of life. In clinical trials, a 5 point change in the MCS and PCS are regarded as clinically relevant, whereas statistical significance can be reached with changes in 1-2 points in the scores.

### Objective measurements

Pre-bronchodilator lung function (forced expiratory volume in one second (FEV_1_) and forced vital capacity (FVC)) and BHR were measured by standard methods [21]. BHR was defined as a 20% reduction in FEV_1 _(PD_20_) after methacholine challenge, with a maximum cumulative methacholine dose of 2 mg [20]. COPD was defined and classified according to the Global initiative for Obstructive Lung Disease (GOLD) [25], i.e FEV_1_/FVC < 0.70. Because the number of subjects with FEV_1 _< 50% predicted were small (COPD GOLD stage III and IV), these were grouped together. Specific IgE levels were measured with the Pharmacia CAP System [26], and atopy was defined as assay results > 0.35 kU/L for any of the following allergens: house dust mite (*Dermatophagoides Pteronyssinus)*, timothy grass, cat and mould (*Cladosporium Herbarum)*.

### Statistical analyses

The statistical software Stata version 10.1 was used for the statistical analyses [27]. The bivariate relationships between the outcome variables (PCS and MCS) and the respiratory symptoms were described by means and standard deviations and tested with Kruskal Wallis and Wilcoxon rank-sum tests. Nonparametric test were used due to skewed distributions of the SF-36 scores. The relationship between the SF-36 scores and each of the symptom variables were investigated in robust multiple linear regression models, with adjustments for age, gender, smoking, occupation, body mass index (BMI), comorbidity, and study centre. Stratified analyses were performed with respect to diagnosis (neither asthma nor COPD; and asthma and/or COPD). Respiratory symptoms often occur together, causing problems of co-linearity if included together, in a mutually adjusted model. Therefore, we used exploratory factor analysis, where symptoms with high level of covariance were summarized in a factor. This yields an estimate of the association between having one of the symptoms in the factor, regardless of which, and HRQoL. The factors substituted the observed symptom variables in multiple linear regression analysis with respect to the SF-36 scores, adjusted for atopy, BHR, and FEV_1 _in addition to age, gender, smoking, occupation, BMI, comorbidity, and study centre.

## Results

Demographic details of the study population (ECRHS II) are given in table 1. In bivariate analyses, both PCS and MCS varied significantly with presence of asthma, gender, age, smoking, occupation, BMI, comorbidity, BHR, but not with atopy. PCS varied with COPD stage, while MCS did not.

**Table 1:** Charachteristis of subjects in ECRHS II, and mean physical component summar (PCS) and mental component summary (MCS) values according to the subjects characteristics.

			PCS			MCS		
		n	Mean	(SD)	^p^	Mean	(SD)	^p^
**COPD**								
	GOLD stage I	236	48.7	(9.9)	< 0.001	49.5	(10.4)	0.406
	GOLD stage II	90	46.1	(10.0)		49.6	(10.9)	
	GOLD stage III/IV	10	34.6	(11.7)		54.9	(7.4)	
**Asthma**								
	No	5368	50.5	(8.8)	< 0.001	50.2	(10.4)	< 0.001
	Yes	598	45.6	(12.0)		48.1	(12.4)	
**Gender**								
	Female	3075	49.5	(9.8)	< 0.001	48.9	(11.3)	< 0.001
	Male	2934	50.5	(8.8)		51.2	(9.7)	
**Age in tertiles**								
	28-39 years	2000	51.3	(8.3)	< 0.001	49.6	(10.7)	< 0.001
	39.1-47 years	2021	50.2	(8.3)		49.6	(10.8)	
	47.1-56.5 years	1988	48.4	(10.3)		50.8	(10.4)	
**Smoking**								
	Never	2541	50.4	(9.3)	< 0.001	50.6	(10.3)	< 0.001
	Ex	1678	50.2	(9.0)		50.3	(10.1)	
	Current	1737	49.3	(9.7)		48.8	(11.5)	
**Occupation**								
	Managers and professionals	1829	51.2	(8.5)	< 0.001	50.0	(10.1)	< 0.001
	Technicians	1115	50.9	(8.3)		50.3	(10.3)	
	Other non manual	1492	49.7	(9.6)		49.4	(11.3)	
	Manual	1185	48.6	(10.0)		51.1	(10.3)	
	Non classified	388	47.6	(11.1)		48.4	(11.9)	
**Body Mass Index**								
	< 20	2739	51.3	(8.4)	< 0.001	49.5	(10.7)	< 0.001
	20-24.9	253	50.6	(9.3)		47.8	(11.3)	
	25-29.9	2106	49.4	(9.4)		50.9	(10.1)	
	30+	773	47.0	(11.0)		50.5	(11.4)	
**Comorbidity**								
	No	2750	52.0	(7.5)	< 0.001	51.3	(9.2)	< 0.001
	Yes	1996	47.8	(10.7)		48.0	(12.0)	
**Atopy**								
	No	3978	49.9	(9.5)	0.359	49.7	(10.9)	0.064
	Yes	1565	50.2	(9.1)		50.7	(9.9)	
**Bronchial hyperresponsiveness**								
	No	3961	50.9	(8.6)	< 0.001	50.3	(10.3)	< 0.001
	Yes	577	48.9	(9.9)		48.0	(11.8)	

All the respiratory symptoms were significantly related to both PCS and MCS, the scores being lower in subjects reporting symptoms (table 2). Breathlessness at rest was the symptom related to the lowest score, both for PCS and MCS. The least impairment in the scores was observed in subjects with cough in the night. A similar pattern of score decrement was observed in multivariate analyses, adjusting for age, gender, smoking, comorbidity, occupation, BMI, and study centre (table 3). The impairment in HRQoL associated with the symptoms was more pronounced for PCS than for MCS.

**Table 2:** Mean (Standard Deviation) physical component summary (PCS) and mental c summary (MCS) in subjects with and without respiratory symptoms in the ECRHS II.

			PCS			MCS		
Symptom			No	Yes		No	Yes	
	*No* *n*	*Yes* *n*	Mean(SD)	Mean(SD)	*p*	Mean(SD)	Mean(SD)	*p*
Wheezing	*4690*	*1317*	50.9(8.5)	46.9(8.5)	*	50.4(10.3)	48.5(11.8)	*
Wheezing and breathlessness	*5311*	*668*	50.6(8.8)	45.4(11.7)	*	50.3(10.4)	47.8(12.1)	*
Wheezing without a cold	*5199*	*761*	50.6(8.8)	45.4(11.7)	*	50.3(10.4)	48.3(11.9)	*
Woken up by tightness in chest	*5228*	*775*	50.7(8.6)	45.3(12.1)	*	50.6(10.2)	46.0(12.5)	*
Breathlessness at rest	*5659*	*339*	50.3(8.9)	44.5(13.3)	*	50.3(10.3)	44.8(13.6)	*
Breathlessness after exercise	*4948*	*1049*	50.9(8.5)	45.7(11.6)	*	50.7(10.1)	46.8(12.3)	*
Breathlessness at night	*5645*	*350*	50.3(9.0)	45.3(12.1)	*	50.2(10.5)	47.2(12.2)	*
Cough at night	*4251*	*1754*	50.9(8.5)	47.9(10.6)	*	50.5(10.3)	48.9(11.2)	*
Cough in the morning	*5258*	*738*	50.5(9.0)	46.9(10.7)	*	50.4(10.4)	47.3(11.7)	*
Cough in the winter	*5233*	*760*	50.6(8.8)	46.0(11.5)	*	50.4(10.2)	47.2(12.8)	*
Chronic chough	*5557*	*404*	50.4(8.9)	45.1(12.2)	*	50.2(10.4)	47.1(12.8)	*
Phlegm in the morning	*5172*	*816*	50.5(8.9)	46.9(11.1)	*	50.3(10.5)	48.2(11.6)	*
Phlegm in the winter	*5342*	*601*	50.5(8.9)	46.0(11.7)	*	50.2(10.5)	48.1(11.8)	*
Chronic phlegm	*5539*	*395*	50.3(9.0)	46.1(12.1)	*	50.1(10.5)	48.3(11.9)	*

**Table 3:** Association (coefficient and 95% confidence interval) of the physical component summary (PCS) and the mental component summa (MCS) with persistent* respiratory symptoms.

	PCS		MCS	
	coef	95% CI	coef	95% CI
Persistent symptoms				
Wheezing	-4.06	(-4.95, -3.16)	-2.45	(-3.41, -1.49)
Wheezing and breathlessness	-6.08	(-7.51, -4.65)	-2.19	(-3.66, -0.72)
Wheezing without a cold	-4.13	(-5.43, -2.84)	-1.88	(-3.14, -0.61)
				
Woken up by tightness in chest	-7.15	(-8.65, -5.65)	-4.02	(-5.51, -2.52)
Breathlessness at rest	-8.05	(-11.18, -4.93)	-3.81	(-6.68, -0.94)
Breathlessness after exercise	-5.93	(-7.11, -4.75)	-2.92	(-4.12, -1.72)
Breathlessness at night	-5.68	(-7.91, -3.45)	-0.78	(-2.95,1.40)
				
Cough at night	-3.68	(-4.46, -2.91)	-2.07	(-2.92, -1.21)
Cough in the morning	-3.68	(-4.89, -2.46)	-2.04	(-3.31, -0.76)
Cough in the winter	-4.74	(-6.05, -3.43)	-4.08	(-5.50, -2.67)
Chronic chough	-4.83	(-6.77, -2.90)	-3.96	(-6.01, -1.90)
				
Phlegm in the morning	-3.92	(-5.04, -2.80)	-2.04	(-3.21, -0.86)
Phlegm in the winter	-4.41	(-5.90, -2.93)	-2.80	(-4.33, -1.28)
Chronic phlegm	-2.64	(-4.42, -0.86)	-2.99	(-5.03, -0.94)

*Persistent meaning presence of the symptom both in ECRHS I and II. Coefficients give deviations from mean score compared to never having had the symptom. Adjusted for age, gender, smoking, comorbidity, occupation, BMI, and study centre.

All the symptoms except breathlessness at night were related to MCS. Breathlessness at rest and waking up with chest tightness were associated with the greatest HRQoL impairment, both in PCS and MCS (table 3).

The adjusted relationship between the symptoms and the HRQoL scores were further explored by analyses stratified by asthma and/or COPD (additional file 1). The relationship between wheeze and breathlessness and the HRQoL scores was similar, whether the subjects had asthma and/or COPD or not, with the exception of breathlessness at rest, which was significantly lower in subjects without asthma and/or COPD compared to those with any of these diagnoses. For cough and phlegm symptoms, the impairment in PCS was somewhat higher in subjects with asthma and/or COPD compared to subjects without these diseases.

Additional analyses assessing the respiratory symptoms-HRQoL relationship in those with asthma and in those with COPD separately showed that these relationships did not differ overtly between subjects with asthma and subjects with COPD (data not shown).

The results of the factor analysis are shown in table 4. Two factors were retained. Symptoms indicated in questions 1-6 (appendix 1) correlated highly and founded the basis for the factor named "wheeze or breathlessness", while questions 7-11 formed a factor for "chronic cough or phlegm". Table 4 displays the results of two multiple linear regression models with respect to PCS and MCS, with the two symptom factors as explanatory variables. Presence of symptoms related to "wheeze or breathlessness" was associated with almost twice the PCS impairment compared to presence of symptoms related to "chronic cough or phlegm". The first model was adjusted for age, gender, smoking, occupation, BMI, comorbidity, and study centre. The second was also adjusted for atopy, BHR, and FEV_1_. The coefficients for the symptom factors did not change significantly when adjusted for atopy, BHR, and FEV_1_.

**Table 4:** Association (coefficient and 95% confidence interval) of the respiratory symptom factors with the physical component summary (PCS) and the mental component summary (MCS) in subjects with neither asthma nor COPD.

	PCS				MCS			
	Coeff.	95% CI		p	Coeff.	95% CI		p
*Model 1^#^*								
Wheezing/breathlessness	-1.88	(-2.24,	-1.52)	< 0.001	-1.57	(-1.95,	-1.18)	< 0.001
Cough/phlegm	-1.04	(-1.37,	-0.71)	< 0.001	-0.92	(-1.28,	-0.55)	< 0.001

*Model 2^##^*								
Wheezing/breathlessness	-1.85	(-2.28,	-1.41)	< 0.001	-1.40	(-1.88,	-0.92)	< 0.001
Cough/phlegm	-0.96	(-1.35,	-0.57)	< 0.001	-0.94	(-1.36,	-0.52)	< 0.001

^# ^Adjusted for age, gender, smoking, occupation, body mass index, comorbidity, and centre.

^## ^Adjusted for age, gender, smoking, occupation, body mass index, comorbidity, centre, atopy, bronchial hyperresponsiveness, and FEV_1_

## Discussion

To our knowledge, this is the first international general population study assessing the association between respiratory symptoms and HRQoL. The association between respiratory symptoms and HRQoL did not depend on having a specific diagnosis of asthma or COPD, as the association was similar in subjects with and without these diseases. Furthermore, in subjects without asthma and/or COPD, the impairment in HRQoL associated with the respiratory symptoms was neither explained by atopy, BHR, nor impairment of lung function.

### HRQoL and other diseases

One of the advantages of using a general HRQoL questionnaire like the SF-36 is that it allows for comparison between different populations. In a Norwegian study where the HRQoL measured by SF-36 of patients with rheumatoid arthritis, epilepsy, angina pectoris, asthma and COPD were compared, and the patients with COPD and rheumatoid arthritis had the lowest physical scores [28]. The mean PCS of patients with COPD was 45, in other words in the same magnitude as the score decrements as the respiratory symptoms investigated in the present study. In a German study evaluating the HRQoL of patients in general practices, the adjusted mean PCS decrements of patients with COPD and asthma were 4.2 points, also comparable to the impairment of PCS scores we found in our study [29]. Our study elaborates on the association between respiratory disease and HRQoL by highlighting the importance of respiratory symptoms, and not only diagnosed disease in this association.

Chronic respiratory symptoms can be caused by other conditions than asthma and COPD. Obesity, heart diseases, hypertension with congestive heart failure, and cancer with metastasis to the lungs can have similar symptomatology. In the current study, both BMI and comorbidities were included as adjustment variables in the analyses. Of the 6009 subjects, 45.8% had no comorbidities 33.2% had one or more, and in 21.0% data on comorbidities were missing. To evaluate if the observed associations between respiratory symptoms and HRQoL could be due to residual confounding by comorbidity, the analyses presented in additional file 1 were also conducted in subjects without comorbidities. In this subgroup, there were still significant associations between respiratory symptoms and HRQoL, both in subjects with and without asthma and/or COPD (data not shown).

### HRQoL, COPD, and asthma

In the current analyses, we chose to join self-reported asthma and spirometrically defined COPD in one group. Firstly, aging is an established risk factor for COPD [17,30,31]. The risk of developing COPD is substantially higher in subjects over 45 years of age [17] compared to younger subjects, and the majority of our study population was under 45 years. Secondly, among our study participants, 336 subjects (5.6%) had airway obstruction measured with pre-bronchodilatator spirometry, and the majority of these had FEV_1 _> 80% of predicted. Most likely, a large number of these subjects have reversible airway obstruction [32], indicating asthma and not COPD. Thirdly, there where 102 subjects that reported ever having had asthma in addition to having FEV_1_/FVC < 0.70. Thus, in a population study as this classification error between asthma and COPD is difficult to avoid. Since the main focus of the study was the subjects without obstructive lung disease, we chose to group asthma and COPD together.

A priori, respiratory symptoms without a known cause will probably be more worrying than symptoms with a recognized pathological origin. A study of asthma and COPD patients in the Netherlands showed that knowledge about both asthma and COPD was related to better adaptation to these diseases [33]. Knowledge of disease is likely to vary in the group of asthma and/or COPD in our study, since asthma was self-reported and COPD was based on spirometric measurements of airflow limitation. To account for an eventual positive effect of knowledge of disease we conducted analyses stratified by asthma and COPD. However, the HRQoL scores did not differ significantly between the subjects with self-reported asthma and respiratory symptoms and the subjects with spirometrically confirmed COPD and respiratory symptoms.

Some symptoms had a greater impact on HRQoL than other symptoms. Subjects with breathlessness without asthma and/or COPD had lower both PCS and MCS than subjects with asthma and/or COPD. It could be that breathlessness indicated by subjects without asthma or COPD represents a different sensation than in subjects with asthma and/or COPD; dyspnoea is a term describing many sensations [34], and the underlying mechanisms of dyspnoea are many [35].

Subjects with cough/phlegm symptoms and asthma/COPD had lower PCS than subjects with these symptoms and neither asthma nor COPD. This difference might be explained by the frequency and intensity of these symptoms, which most likely are higher in subjects with asthma and/or COPD. The symptom questionnaire did not assess symptom frequency and intensity, so this remains unsolved. COPD is a heterogeneous disease, and the classical way of describing the different phenotypes have been through the Snider venn diagram. Snider described the variety of obstructive lung disease is through the phenotype with asthma, the one with chronic bronchitis, the one with emphysema, and varying degrees of overlap between the three phenotypes. The hallmark symptoms of the chronic bronchitis phenotype often are cough and phlegm. The decreased PCS accompanying the cough and phlegm in our analyses of subjects with asthma and/or COPD may indicate that subjects with the chronic bronchitis phenotype have lower HRQoL than the asthma and emphysema phenotypes.

### Objective measurements

When atopy, BHR and FEV_1 _was included in the regression model with the symptom factors, the regression coefficients did not change overtly. Garcia-Marcos et al evaluated HRQoL in asthmatic children and found that atopy was not a risk factor for decreased HRQoL [36]. We have found no previous general population studies investigating BHR in the context of HRQoL. Better lung function was related to higher HRQoL as found in another study [31], but the difference in FEV_1 _associated with one point change in the PCS score was more than 1000 ml. In other words, a change in PCS score barely statistically significant corresponded to five times the FEV1 change regarded as clinically relevant spirometrically.

## Conclusion

Respiratory symptoms are among the more common health problems in the general population. Our results have shown that otherwise healthy young adults reporting respiratory symptoms have a relevant impairment in quality of life, which is not restricted to those with asthma or COPD and cannot be explained by atopy, reduced lung function, or BHR. Understanding the nature of these symptoms and the implications for diagnosis, treatment and prevention, remains a relevant challenge. Furthermore, our study suggests that it is important to adjust for respiratory symptoms, and not only respiratory disease or other comorbidities when HRQoL is evaluated.

## Competing interests

Authors MVA, TMLE, EP, ERO, CS, VS, IP, JMA, and BL have no conflicts of interest.

Within the last 5 years MVA, TMLE, and PSB have received sponsorship for travel and accommodation to the ATS conference from GlaxoSmithKline, and TMLE has received grant monies from AstraZeneca. PSB has received 2000 € in lecture fees from GlaxoSmithKline and 1000 € in lecture fees from AstraZeneca during the last 5 years. PSB acts as a principle investigator in the ECLIPSE study. None of this is related to the present study.

## Authors' contributions

The presented work is carried out in collaboration between all authors. JMA and MVA defined the research theme. JMA, CS, ERO, and BL designed the data collection. EP and MVA conducted the statistical analyses. Results interpreted by JMA, EP, and MVA. All authors contributed to the drafting and critical revising of the manuscript.

## Appendix 1

Wheezing or whistling in your chest in the last 12 months

If yes to 1, breathless when wheezing sound present

If yes to 1, wheezing and whistling without cold

Woken up with a feeling of chest tightness in the last 12 months

Attack of short breath after activity last 12 months

Attack of shortness of breath at rest in the last 12 months

Woken by attack of shortness of breath in the last 12 months

Woken by attack of coughing in the last 12 months

Usually cough first thing in the morning in winter?

Usually cough in day or night during winter?

7.1 Cough like this for 3 months a year?

Bring up phlegm from your chest in the morning in the winter?

Bring up phlegm during day or night in the winter?

Bring up phlegm most days for three months each year?

Ever had asthma?

## Appendix 2

Suffer from long term limiting illness

Suffer with arthritis

Suffer with hypertension-high blood presure

Suffer with deafness

Suffer with varicose veins

Suffer from cataracts

Suffer from heart disease

Suffer from depression

Suffer from diabetes

Suffer from migraine/recurrent headaches

Have cancer

Had a stroke

## Supplementary Material

Additional file 1 **Association (coefficient and 95% CI) of the physical component summary (PCS) and the mental component summary (MCS) with respiratory symptoms in the ECRHS II by asthma and/or COPD, adjusted for age, gender, smoking, comorbidity, and country**. Click here for file
