# Supplementary material for: Respiratory symptoms in adults are related to impaired quality of life, regardless of asthma and COPD: results from the European community respiratory health survey
Source: Health Qual Life Outcomes. 2010 Sep 27;8:107. doi: 10.1186/1477-7525-8-107 (PMC2954977; doi:10.1186/1477-7525-8-107)
Supplement: Additional file 1 — Association (coefficient and 95% CI) of the physical component summary (PCS) and the mental component summary (MCS) with respiratory symptoms in the ECRHS II by asthma and/or COPD, adjusted for age, gender, smoking, comorbidity, and country. [file 1477-7525-8-107-S1.DOC]

| **Additional file 1. Association (coefficient and 95% CI) of the physical component summary (PCS) and the mental component summary (MCS) with respiratory symptoms in the ECRHS II by asthma and/or COPD, adjusted for age, gender, smoking, comorbidity, and country.** | | | | | | | | | | | | |
| --- | --- | --- | --- | --- | --- | --- | --- | --- | --- | --- | --- | --- |
|
|
|
|
|
|
|  | **PCS** | | | | | | **MCS** | | | | | |
|  | **No asthma, no COPD** | | | **Asthma and/or COPD** | | | **No asthma, no COPD** | | | **Asthma and/or COPD** | | |
|  | **N= 5, 095** | | | **N= 905** | | | **N= 5, 095** | | | **N= 905** | | |
|  | coef | 95% CI | | coef | 95% CI | | coef | 95% CI | | coef | 95% CI | |
| SYMPTOMS |  |  |  |  |  |  |  |  |  |  |  |  |
| Wheezing | -3.21 | (-4.05 , | -2.37) | -3.38 | (-4.86 , | -1.90) | -1.77 | (-2.69 , | -0.86) | -1.45 | (-2.99 , | 0.10) |
| Wheezing and breathlessness | -3.94 | (-5.28 , | -2.59) | -4.19 | (-5.73 , | -2.65) | -2.83 | (-4.20 , | -1.45) | -1.78 | (-3.33 , | -0.23) |
| Wheezing without a cold | -3.42 | (-4.62 , | -2.22) | -2.76 | (-4.31 , | -1.22) | -2.95 | (-4.24 , | -1.66) | -0.71 | (-2.23 , | 0.81) |
|  |  |  |  |  |  |  |  |  |  |  |  |  |
| Woken up by tightness in chest | -4.71 | (-5.84 , | -3.59) | -5.00 | (-6.71 , | -3.29) | -4.04 | (-5.17 , | -2.90) | -3.00 | (-4.74 , | -1.26) |
| Breatlessness at rest | -6.13 | (-8.17 , | -4.09) | -2.70 | (-4.88 , | -0.52) | -5.71 | (-7.77 , | -3.65) | -2.62 | (-4.89 , | -0.35) |
| Breathlessnss after exercise | -4.14 | (-5.06 , | -3.22) | -4.62 | (-6.08 , | -3.15) | -2.79 | (-3.77 , | -1.80) | -1.79 | (-3.39 , | -0.18) |
| Breathlessness at night | -4.55 | (-6.35 , | -2.74) | -4.20 | (-6.31 , | -2.09) | -2.43 | (-4.16 , | -0.70) | -2.38 | (-4.64 , | -0.11) |
|  |  |  |  |  |  |  |  |  |  |  |  |  |
| Cough at night | -2.74 | (-3.37 , | -2.10) | -2.18 | (-3.72 , | -0.65) | -0.98 | (-1.67 , | -0.29) | -1.72 | (-3.31 , | -0.13) |
| Cough in the morning | -3.02 | (-4.00 , | -2.04) | -3.27 | (-5.06 , | -1.49) | -2.37 | (-3.44 , | -1.31) | -2.26 | (-4.15 , | -0.37) |
| Cough in the winter | -3.24 | (-4.25 , | -2.23) | -5.14 | (-6.91 , | -3.38) | -3.17 | (-4.36 , | -1.98) | -2.22 | (-4.03 , | -0.41) |
| Chronic cough | -3.98 | (-5.45 , | -2.50) | -6.58 | (-8.91 , | -4.26) | -3.16 | (-4.75 , | -1.57) | -1.19 | (-3.56 , | 1.18) |
|  |  |  |  |  |  |  |  |  |  |  |  |  |
| Phlegm in the morning | -2.56 | (-3.49 , | -1.63) | -5.02 | (-6.86 , | -3.18) | -2.04 | (-3.04 , | -1.03) | -1.97 | (-3.78 , | -0.16) |
| Phlegm in the winter | -2.92 | (-4.06 , | -1.78) | -5.35 | (-7.34 , | -3.36) | -1.75 | (-2.95 , | -0.56) | -1.99 | (-3.92 , | -0.06) |
| Chronic phlegm | -2.67 | (-4.11 , | -1.24) | -5.11 | (-7.52 , | -2.69) | -1.89 | (-3.36 , | -0.41) | -1.07 | (-3.18 , | 1.04) |
